# Streamlining postprocedural care after transcatheter edge-to-edge repair

**DOI:** 10.1186/s12872-026-05524-2

**Published:** 2026-02-05

**Authors:** Jakob Christoph Voran, Jan Baucks, Violetta Catania, Peter Bramlage, Derk Frank, Felix Kreidel

**Affiliations:** 1 Department of Internal Medicine III, Cardiology and Critical Care, University Hospital Schleswig-Holstein, Campus Kiel, Arnold-Heller-Strasse 3, Kiel, 24105 Germany; 2 DZHK (German Centre for Cardiovascular Research), Partner site Hamburg/Kiel/Lübeck, Potsdamer Strasse 58, Berlin, 10785 Germany; 3 Institute for Pharmacology and Preventive Medicine, Bahnhofstrasse 20, Cloppenburg, 49661 Germany

**Keywords:** Mitral valve repair, Tricuspid valve repair TEER, Intensive care, Postprocedural care, Quality of care

## Abstract

**Background:**

Patients undergoing transcatheter edge-to-edge repair (TEER) of the mitral or tricuspid valve are predominantly elderly and exhibit high rates of frailty. The procedure regularly involves general anaesthesia, necessitating the monitoring of patients on an intensive or intermediate care unit (ICU/ImCU). This study aimed to investigate the outcome of patients being admitted to a general ward as opposed to an ICU/ImCU for postprocedural care after TEER.

**Methods:**

This is a retrospective study analysing the course of 209 patients that underwent TEER at a university hospital centre in Germany from January 2022 to February 2024. Patients were assigned to either intended postprocedural care at ICU/ImCU (*n* = 113) or streamlined care (*n* = 95) based on a cut-off date unrelated to this study.

**Results:**

In comparison to the ICU/ImCU group, patients in the streamlined group exhibited a significantly reduced total hospital stay (median 4 [IQR 3, 9] vs. 3 [IQR 3, 5] days, *p* = 0.009) and a reduced postprocedural length of stay without an elevated rate of unplanned ICU/ImCU admissions. Postprocedural care in the streamlined group was not associated with a higher rate of complications nor a higher mortality rate at 30 days (0.9% vs. 2.2%, *p* = 0.6), six months (10.1% vs. 4.7%, *p* = 0.2) or 12 months (16.0% vs. 12.0%, *p* = 0.4).

**Conclusions:**

The transition of postprocedural care from the ICU/ImCU to a general ward was found to be safe, reduced length of hospital stay and spared the utilization of ICU/ImCU resources. This encourages for randomised clinical trials to further advance the streamlined care of these patients.

## Introduction

Transcatheter edge-to-edge repair (TEER) has emerged as a safe and potentially effective therapy in patients with severe mitral regurgitation (MR) and tricuspid regurgitation (TR) with prohibitive surgical risks [1, 2]. Although it has become a routine alternative to cardiac surgery, a heterogeneity of clinical practices persist in the management of patients following TEER. Due to the fact that TEER is mainly performed under general anaesthesia (GA) and patients frequently present with multiple comorbidities, postprocedural patient care largely involves monitoring in either an intensive care unit (ICU) or intermediate care unit (ImCU) prior to transferring the patient to a general cardiology ward for further recovery and discharge [3]. This, as a result, imposes a restriction on capacity, contributing to increased waiting time delays and resulting in an increase in length of stay (LoS) and associated costs.

In Germany, there is currently no standardization for patients undergoing either mitral or tricuspid TEER procedures with regards to care post-procedure, although ICU or ImCU monitoring is commonly preferred. Several studies have assessed the use of deep sedation/conscious sedation (DS/CS) rather than GA to reduce the duration of ICU stay after mitral TEER (M-TEER) and revealed conflicting results [4–6]. Recently, Gröger et al. evaluated the use of a dedicated valve unit instead of ICU/ImCU in a retrospective analysis of 624 patients undergoing M-TEER using GA [7]. The authors demonstrated that the immediate admission to the valve unit, as opposed to primary ICU/ImCU resulted in a reduced total hospital LoS (6 vs. 7 days, *p* < 0.001) and a lower incidence of infections (2.9 vs. 7.7%, *p* = 0.008), while patient safety was uncompromised (in-hospital mortality: 0.6% in the valve unit group and 1.3% in the ICU group, *p* = 0.41) [7]. As for tricuspid TEER (T-TEER), data on post-procedure practice after GA is very scarce. In a retrospective study conducted at three centers comparing DS (*n* = 40) with GA (*n* = 64) T-TEER, the total hospital LoS was shorter (6 vs. 8 days, *p* = 0.011) with 90% of DS patients going to the general ward (GW) after observation in the recovery area, whereas all patients in the GA group were transferred to either ICU or ImCU [8]. 

Given the anticipated growth in structural heart procedures, coupled with the paucity of studies evaluating the impact of avoiding ICU/ImCU facilities after TEER on outcomes and resource utilization, there is a clear need to evaluate the effectiveness of a less intensive approach. This would help to guide rational resource utilization in the current economic climate, which is characterized by staff constraints and shortages. To this end, we aimed to streamline postprocedural care after M-/T-TEER by transferring patients to the GW without the interim need for an ICU/ImCU admission (Central Figure).

## Methods

### Study design and objectives

This observational, retrospective study evaluated consecutive patients undergoing TEER for MR and TR at a single institution in Germany. The primary objective of this study was to demonstrate safety of streamlined postprocedural care after M-/T-TEER by avoiding the requirement for ICU/ImCU.

### Ethics

The study was conducted in accordance with the principles stated in the World Medical Association’s Declaration of Helsinki. Ethical approval was obtained from the local ethics committee (Ethics Comitee of the Medical Faculty of the Christian-Albrechts-University Kiel; AZ 506/22). All patients consented to share follow-up information by signing a broad consent form at admission.

### Standard operating procedure – streamlined postprocedural care

The Standard Operating Procedure that came into effect on 7th March 2023 established the protocol for postprocedural care following interventions involving GA in our catheter laboratory. In order to be considered for monitoring in the holding area, the following criteria had to be met: spontaneous breathing, complete awakening, positive protective reflexes, haemodynamic stability, secure venous and no arterial access. In the event that these criteria are not met, the patient was admitted to the ImCU. Preprocedural workup and intraoperative management of patients did not differ between the two study groups.

The monitoring in the holding area included monitoring of oxygen saturation *via* finger clip, electrocardiography, non-invasive blood pressure (at five-minute intervals during the initial 30 min), control of access site 5, 15 and 30 min prior to admittance to GW and documentation of monitoring at 15-minute intervals or in the event of a deviation from the norm. The monitoring was overseen by a medical doctor.

Prior to admission to the GW, patients had to demonstrate stable hemodynamics, unrestricted vigilance, and unremarkable puncture sites. Subsequent to admission, vigilance and hemodynamics must be re-evaluated.

### Patient population

Consecutive patients undergoing TEER for MR or TR as per the current approved indication after Heart Team discussion and agreement and with written informed consent were included in the study. Patients with an emergency indication or who were treated at ICU or ImCU prior to TEER were excluded. The cut-off date between the groups was the 7th March 2023. All patients underwent general anesthesia (regardless of ICU/ImCU admission before the cut-off date or transfer to the recovery area/GW (streamlined cohort)).

### Study outcomes

Clinical follow-up was performed at discharge, 30 days, six months and 12 months after the procedure. The primary safety endpoint was defined as freedom from cardiovascular mortality and unplanned re-admission for either heart failure decompensation or procedure-related complications or unplanned treatment encounter within 30 days of the TEER procedure. Secondary endpoints included postprocedural length of stay (LoS) in ICU, ImCU, and general cardiology ward; unplanned in-hospital transfer to ICU/ImCU; all-cause and cardiovascular mortality at 30 days; major adverse events at 30 days, which were defined as major bleeding; pericardial effusion/cardiac tamponade requiring pericardiocentesis; the need for blood transfusion and major access site and vascular complications; mitral and/or tricuspid regurgitation severity assessed by echocardiography at discharge, as compared to baseline; and unplanned further mitral/tricuspid intervention within 30 days of the procedure. All safety endpoints were defined in accordance to the Mitral Valve Academic Research Consortium (MVARC) criteria with the exception of bleeding events and technical success, which were defined using the Valve Academic Research Consortium 3 (VARC-3) bleeding scale or the Tricuspid Valve Academic Research Consortium (TVARC) Criteria [9–11]. 

### Statistical analysis

Continuous variables were presented as mean ± standard deviation (SD) or median and interquartile range (IQR), according to the skewness of the data. The Kolmogorov-Smirnov test was utilized to test for normal distribution. Categorical variables were reported as frequencies and percentages. Student’s t-test or Mann-Whitney U test were utilized for comparing continuous variables, and Pearson’s chi-square or Fisher’s exact test were utilized for categorical variables, as appropriate. A p-value of < 0.05 was considered statistically significant. All analyses were performed using R version 4.4.1 (14th June 2025), packages “tidyverse”, “readxl”, survival”, “ggsurvfit”, “gtsummary” [12–16].

## Results

**Fig. 1:**
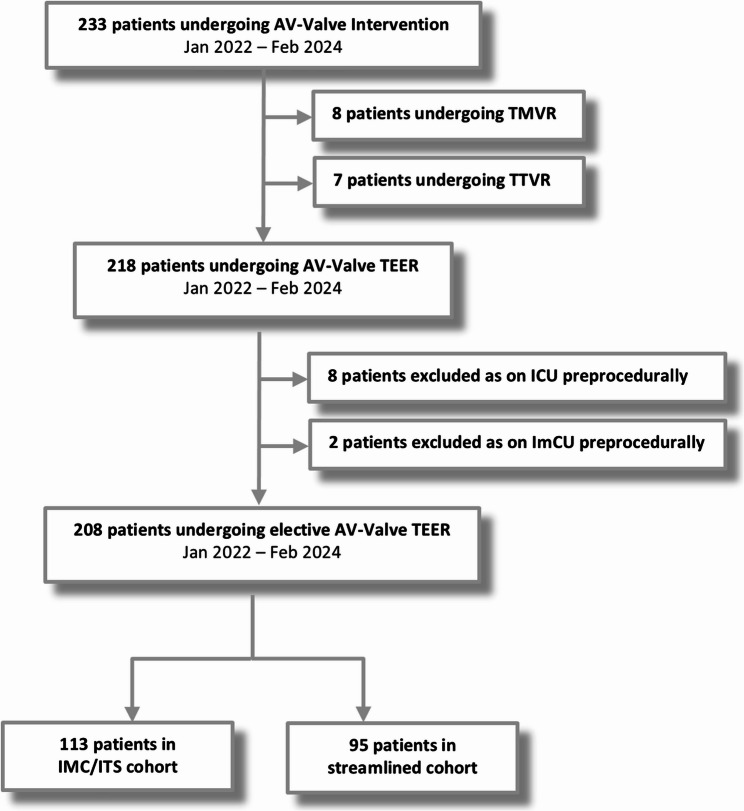
Study flowchart. Legend: AV, atrioventricular; ICU, intensive care unit; ImCU, intermediate care unit; TEER, transcatheter edge-to-edge repair; TMVR, transcatheter mitral valve replacement; TTVR, transcatheter tricuspid valve replacement

Between January 2022 and February 2024, a total of 233 patients underwent atrioventricular valve intervention at our institution. Eight patients underwent transcatheter mitral valve replacement and seven transcatheter tricuspid valve replacement and were excluded from the study (Fig. 1). Out of 218 patients undergoing TEER, ten patients were further excluded due to already being on ICU/ImCU prior to the procedure. As a result, a consecutive series of 208 patients were analysed: 113 patients were transferred to the ICU/ImCU, while 95 were transferred to the recovery area/GW (streamlined cohort) following M-/T-TEER.

### Patient characteristics

At baseline, patients in the ICU/ImCU cohort exhibited significantly higher prevalence of New York Heart Association (NYHA) functional class IV compared to those in the streamlined cohort (ICU/ImCU: 20% vs. streamlined: 6.3%, *p* = 0.028). This also applied to the prevalence of diabetes (ICU/ImCU: 25% vs. streamlined: 12%, *p* = 0.014), previous one-chamber (ICU/ImCU: 8.0% vs. streamlined: 3.2%) or two-chamber (ICU/ImCU: 13% vs. streamlined: 7.4%) pacemaker device (*p* = 0.045) and percutaneous coronary intervention (PCI) in the long-term, i.e. >3 months (ICU/ImCU: 64% vs. streamlined: 35%, *p* = 0.001), whereas patients in the streamlined cohort were more frequently hospitalised due to heart failure in the preceding year at least once (streamlined: 45% vs. ICU/ImCU: 33%, *p* < 0.001; Table 1). Furthermore, patients transferred to ICU/ImCU exhibited significantly higher left ventricular (LV) diameters (LV end-diastolic diameter: ICU/ImCU: 53.04 vs. streamlined: 50.18 mm, *p* = 0.011; LV end-systolic diameter: ICU/ImCU: 40.82 vs. streamlined: 38.02 mm, *p* = 0.016) and higher rates of heart failure with reduced ejection fraction (with left ventricular ejection fraction ≤ 40%; ICU/ImCU: 26% vs. streamlined: 14%, *p* = 0.047; Table 2) in comparison to those in the streamlined chohort. No differences were observed in surgical risk, the laboratory values or echocardiographic evaluation of MR/TR between the study groups at baseline, with the exception of larger MR vena contracta width (ICU/ImCU: 5.33 vs. streamlined: 4.48 mm, *p* = 0.007) and an increased TR regurgitant volume (ICU/ImCU: 45.82 vs. streamlined: 36.35 ml, *p* = 0.041) in the ICU/ImCU cohort.

**Table 1:** Patient baseline characteristics

	ICU/ImCU (*n* = 113)	Streamlined (*n* = 95)	*p*-value
Age, years	77.87 ± 10.03	79.28 ± 11.54	0.086
	80.19 [76.00, 84.91]	82.00 [77.00, 85.36]	
Body mass index, kg/m²	25.52 ± 4.95	25.33 ± 5.37	0.4
	25.10 [22.10, 27.80]	24.40 [22.10, 26.60]	
Male sex	61 (54%)	47 (49%)	0.5
NYHA class			**0.028**
I	2 (2.3%)	3 (3.8%)	
II	11 (13%)	17 (21%)	
III	57 (65%)	55 (69%)	
IV	18 (20%)	5 (6.3%)	
EuroSCORE II, %	4.36 ± 3.31	5.98 ± 5.34	0.070
	3.88 [1.94, 5.65]	4.49 [2.33, 7.61]	
6-minute walk test, m	280.32 ± 117.17	243.29 ± 124.24	0.3
	270.00 [200.00, 350.00]	245.00 [150.00,357.50]	
KCCQ-12	44.56 ± 21.52	45.38 ± 25.48	> 0.9
	44.27 [27.08, 60.42]	34.38 [30.47, 64.58]	
Number of heart failure hospitalizations in the preceding year			**< 0.001**
1	37 (33%)	43 (45%)	
> 1	12 (11.0%)	13 (13.6%)	
CPD	21 (19%)	16 (17%)	0.7
Arterial hypertension	83 (75%)	76 (81%)	0.3
Chronic kidney disease			0.3
Grade 1	4 (3.5%)	0 (0%)	
Grade 2	26 (23%)	28 (29%)	
Grade 3	63 (56%)	49 (52%)	
Grade 4	13 (12%)	15 (16%)	
Grade 5	4 (3.5%)	1 (1.1%)	
Diabetes mellitus(Type I, II)	28 (25%)	11 (12%)	**0.014**
Cerebrovascular disease	17 (15%)	11 (12%)	0.5
Stroke	17 (15%)	13 (14%)	0.8
Atrial fibrillation			0.3
paroxysmal	23 (20%)	21 (22%)	
persistent	13 (12%)	18 (19%)	
permanent	49 (43%)	40 (42%)	
Peripheral vascular disease	13 (12%)	13 (14%)	0.6
Coronary artery disease	71 (63%)	55 (58%)	0.5
Prior myocardial infarction			0.6
anterior	4 (3.5%)	2 (2.1%)	
inferior/lateral	6 (5.3%)	7 (7.4%)	
Peripheral oedema*			**< 0.001**
1	15 (13%)	16 (17%)	
2	10 (8.8%)	29 (31%)	
3	4 (3.5%)	3 (3.2%)	
Previous PCI Long-term (> 3 months)	42 (64%)	19 (35%)	**0.001**
Previous PCI Short-term (< 3 months)	26 (39%)	2 (36%)	0.8
Previous CABG	15 (21%)	14 (25%)	0.6
Previous electric device			**0.045**
1-chamber PM/ICD	9 (8.0%)	3 (3.2%)	
2-chamber PM/ICD	15 (13%)	7 (7.4%)	
CRT-P	2 (1.8%)	5 (5.3%)	
CRT-D	3 (2.7%)	9 (9.6%)	
Previous aortic valve surgery			0.6
Repair	0 (0%)	0 (0%)	
Replacement	13 (12%)	9 (9.5%)	
Previous tricuspid valve surgery			0.7
Repair	0 (0%)	1 (1.1%)	
Replacement	1 (0.9%)	0 (0%)	
Previous mitral valve surgery			0.3
Repair	15 (13%)	6 (6.3%)	
Replacement	2 (1.8%)	2 (2.1%)	

Legend: Data are presented as mean ± SD, median [IQR] or n (%)

*Oedema stages are defined as follows: stage 1 = swelling of the feet, stage 2 = swelling of the lower leg, stage 3 = swelling of the whole leg

*CABG* Coronary artery bypass graft, *CPD* Chronic pulmonary disease, *CRT-D* Cardiac resynchronization therapy defibrillator, *CRT-P* Cardiac resynchronization therapy pacemaker, *EuroSCORE* European system for cardiac operative risk evaluation, *ICD* Implantable cardioverter-defibrillator, *ICU* Intensive care unit, *IQR* Inter quartile range, *ImCU* Intermediate care unit, *KCCQ* Kansas City Cardiomyopathy Questionnaire, *NYHA* New York Heart Association, *PCI* Percutaneous coronary intervention, *PM* Pacemaker, *SD* Standard deviation

**Table 2:** Echocardiography and lab parameters

	ICU/ImCU (*n* = 113)	Streamlined (*n* = 95)	*p*-value
Creatinine, µmol/l	150.07 ± 110.57	132.28 ± 58.10	0.6
	121.00 [92.50, 164.00]	111.00 [94.00, 166.00]	
GFR, ml/min/1.73 m²	45.21 ± 20.63	45.95 ± 19.49	0.8
	44.00 [28.00, 59.00]	43.00 [30.00, 60.00]	
Haemoglobin, g/dl	12.04 ± 2.13	12.20 ± 2.13	0.7
	11.90 [10.50, 13.60]	12.40 [10.60, 13.60]	
Troponin T, ng/l	35.58 ± 23.29	37.36 ± 29.53	> 0.9
	28.85 [18.70, 45.30]	28.90 [20.70, 44.10]	
NT-proBNP, ng/l	5,500.56 ± 10,553.05	3,986.48 ± 4,378.26	0.3
	2,728.00 [1,687.00, 4,741.00]	2,744.00 [1,093.00, 5,670.00]	
sPAP, mmHg	44.24 ± 12.43	44.25 ± 13.64	0.8
	42.00 [35.00, 50.00]	42.00 [35.00, 52.00]	
LA volume, ml	108.75 ± 58.46	119.74 ± 69.33	0.13
	100.45 [76.60, 130.00]	113.60 [81.40, 133.10]	
LVEDD, mm	53.04 ± 8.92	50.18 ± 10.17	**0.011**
	51.00 [47.00, 58.00]	48.00 [43.00, 55.50]	
LVESD, mm	40.82 ± 10.33	38.02 ± 12.18	**0.016**
	40.00 [34.00, 45.00]	35.00 [30.00, 45.00]	
LVEDV, ml	125.61 ± 70.69	117.83 ± 64.54	0.3
	117.10 [83.00, 147.50]	99.50 [78.40, 136.10]	
LVESV, ml	70.05 ± 60.45	63.06 ± 53.61	0.2
	55.00 [40.40, 76.60]	48.45 [33.90, 66.25]	
LVEF (calculated), %	47.65 ± 12.58	49.94 ± 10.77	0.3
	50.50 [43.00, 56.00]	52.00 [46.00, 56.00]	
HFrEF			**0.047**
HFrEF	25 (26%)	12 (14%)	
MR aetiology			0.5
Primary	34 (47%)	38 (57%)	
Secondary			
Ventricular	12 (16%)	12 (18%)	
Atrial	24 (33%)	16 (24%)	
Mixed	3 (4.1%)	1 (1.5%)	
MR EROA, cm²	0.35 ± 0.24	0.32 ± 0.20	0.4
	0.31 [0.23, 0.40]	0.31 [0.22, 0.38]	
MR regurgitant volume, ml	39.68 ± 20.88	42.56 ± 23.60	0.3
	36.00 [28.00, 50.20]	40.00 [30.00, 57.00]	
MR vena contracta, mm	5.33 ± 2.02	4.48 ± 2.20	**0.007**
	5.00 [4.00, 6.80]	4.10 [2.90, 5.40]	
MR grade			0.2
< I	4 (3.9%)	2 (2.3%)	
I	24 (24%)	17 (20%)	
II	28 (27%)	25 (29%)	
III	36 (35%)	24 (28%)	
IV	10 (9.8%)	19 (22%)	
MV PG mean, mmHg	2.63 ± 1.09	2.49 ± 1.24	0.2
	2.42 [1.95, 3.15]	2.00 [1.66, 3.00]	
TR aetiology			0.4
Primary	4 (10%)	3 (11%)	
Secondary			
Atrial	18 (45%)	18 (64%)	
Ventricular	16 (40%)	7 (25%)	
Mixed	2 (5.0%)	0 (0%)	
TR EROA, cm²	0.51 ± 0.29	0.43 ± 0.28	0.11
	0.46 [0.33, 0.61]	0.44 [0.19, 0.60]	
TR regurgitant volume, ml	45.82 ± 25.32	36.35 ± 20.95	0.041
	42.75 [29.00, 57.60]	36.50 [22.00, 50.00]	
TR vena contracta, mm	6.70 ± 3.98	6.24 ± 4.19	0.2
	5.70 [4.00, 8.00]	5.00 [3.00, 9.00]	
TR grade			0.12
< I	2 (2.1%)	1 (1.1%)	
I	18 (19%)	29 (33%)	
II	21 (22%)	15 (17%)	
III	40 (41%)	23 (26%)	
IV	11 (11%)	14 (16%)	
V	5 (5.2%)	5 (5.7%)	
TV PG mean, mmHg	1.62 ± 0.83	1.44 ± 0.60	0.4
	1.35 [1.00, 1.95]	1.28 [1.00, 1.72]	
TAPSE, mm	17.56 ± 5.20	18.21 ± 5.24	0.2
	17.00 [14.00, 21.00]	19.00 [16.00, 22.00]	

Legend: Data are presented as mean ± SD, median [IQR] or n (%)

*EROA* Effective regurgitant orifice area, *GFR* Glomerular filtration rate, *HFrEF* Heart failure with reduced ejection fraction, *ICU* Intensive care unit, *ImCU* Intermediate care unit, *IQR* Inter quartile range, *LA* Left atrial, *LVEDD* Left ventricular end-diastolic diameter, *LVEDV* Left ventricular end-diastolic volume, *LVEF* Left ventricular ejection, *LVESD* Left ventricular end-systolic diameter, *LVESV* Left ventricular end-systolic volume, *MR* Mitral regurgitation, *MV* Mitral valve, *NT-proBNP* N-terminal pro B-type natriuretic peptide, *PG* Pressure gradient, *SD* Standard deviation, *sPAP* Systolic pulmonary arterial pressure, *TAPSE* Tricuspid Annular Plane Systolic Excursion, *TR* Tricuspid regurgitation, *TV* Tricuspid valve

### Procedural details

**Fig. 2:**
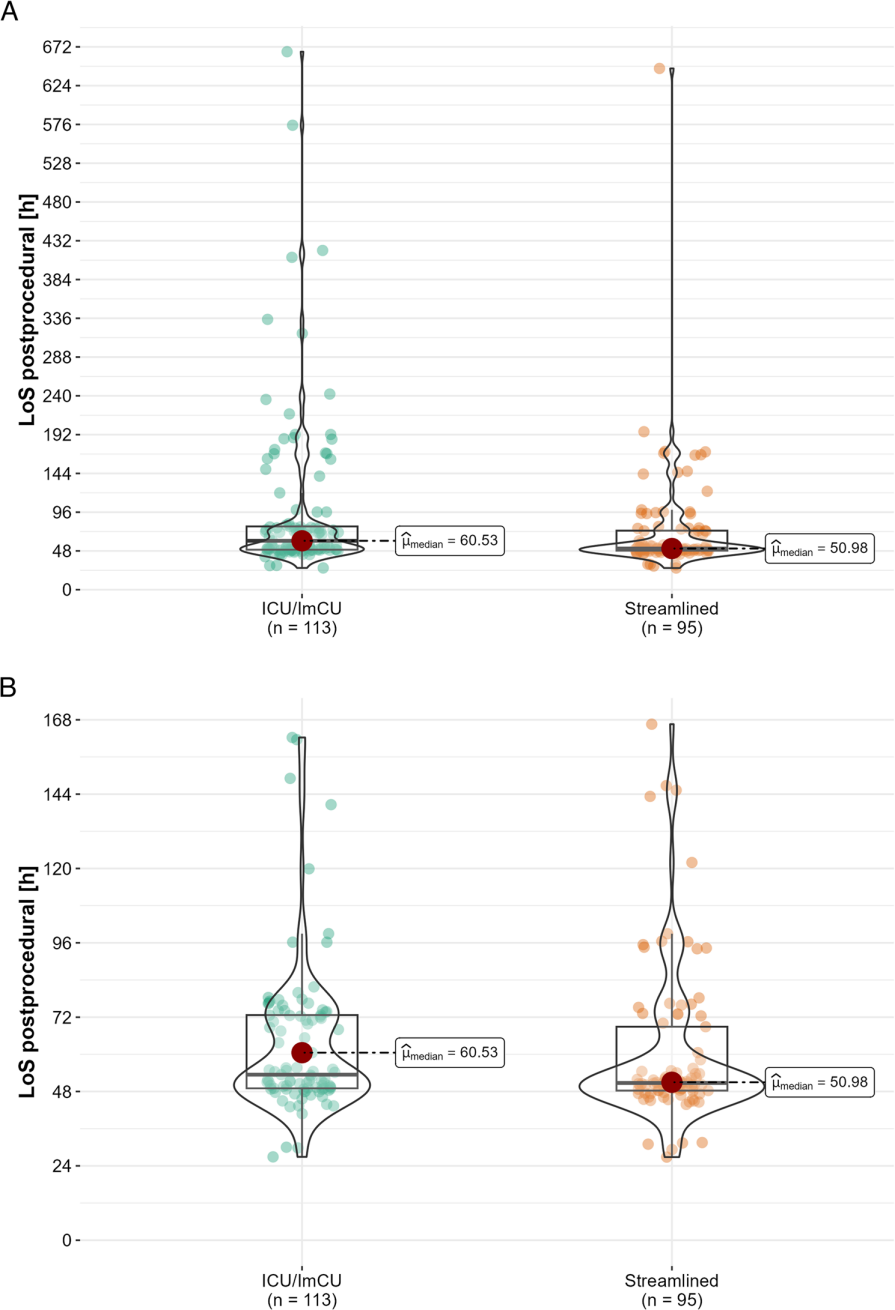
Postprocedural LoS including outliers (**A**) and zoomed (**B**). Legend: LoS, length of stay; h, hours; ICU, intensive care unit; ImCU, intermediate care unit

Patients in the ICU/ImCU and streamlined cohorts equally underwent TEER for MR and TR (*p* = 0.4; Table 3). All patients were treated under GA. No significant disparities in the technical success rates were observed. Patients in the streamlined cohort exhibited a significantly shorter procedure duration (streamlined: median 64.50 [IQR 52.00, 80.00] vs. ICU/ImCU: 77.00 min [IQR 60.00, 103.00], *p* = 0.002) and shorter postprocedural LoS (streamlined: median 2.1 [IQR 2.0, 3.1] vs. ICU/ImCU: 2.5 days [IQR 2.1, 3.3], *p* = 0.011) compared to patients admitted to ICU/ImCU after the procedure (Fig. 2). Total hospital LoS was also significantly shorter in the streamlined group (streamlined: median 3.00 [IQR 3.00, 5.00] vs. ICU/ImCU: 4.00 days [IQR 3.00, 9.00], *p* = 0.009). MR/TR grades at discharge were found to be comparable between the study groups. The discharge rate from the hospital to home was over 90% for both groups (*p* = 0.8).

**Table 3:** Procedural data

	ICU/ImCU (*n* = 113)	Streamlined (*n* = 95)	*p*-value
Treated valve			0.4
mitral valve	73 (65%)	67 (71%)	
tricuspid valve	40 (35%)	28 (29%)	
Device used			0.5
MitraClip	39 (35%)	36 (38%)	
TriClip	25 (22%)	15 (16%)	
PASCAL system	49 (43%)	44 (46%)	
Number of implanted devices			0.3
0	3 (2.7%)	1 (1.1%)	
1	60 (53%)	58 (61%)	
2	46 (41%)	29 (31%)	
3	4 (3.5%)	7 (7.4%)	
Single leaflet device attachment	4 (3.5%)	2 (2.1%)	0.7
Technical success	99 (88%)	84 (88%)	> 0.9
Infection requiring antibiotics	8 (7.1%)	3 (3.2%)	0.2
Delirium	9 (8.0%)	4 (4.2%)	0.3
Periinterventional CPR	0 (0%)	2 (2.1%)	0.2
Procedure time, min	77.00 [60.00, 103.00]	64.50 [52.00, 80.00]	**0.002**
Time on ICU/ImCU, hours	21.32 [13.07, 23.78]	0.00 [0.00, 0.00]	**< 0.001**
Time on general ward, hours	46.13 [30.28, 62.23]	50.75 [47.68, 73.22]	**< 0.001**
Postprocedural LoS, days	2.5 [2.1, 3.3]	2.1 [2.0, 3.1]	**0.011**
Total hospital LoS, days	4.00 [3.00, 9.00]	3.00 [3.00, 5.00]	**0.009**
MR grade at discharge			0.7
< I	6 (5.4%)	6 (6.4%)	
I	78 (70%)	72 (77%)	
II	23 (21%)	13 (14%)	
III	3 (2.7%)	3 (3.2%)	
IV	1 (0.9%)	0 (0%)	
TR grade at discharge			0.9
< I	5 (4.7%)	2 (2.2%)	
I	47 (44%)	45 (48%)	
II	31 (29%)	22 (24%)	
III	17 (16%)	18 (19%)	
IV	6 (5.6%)	5 (5.4%)	
V	1 (0.9%)	1 (1.1%)	
Discharge to			0.8
home	73 (94%)	84 (93%)	
nursing	1 (1.3%)	1 (1.1%)	
geriatrics	1 (1.3%)	3 (3.3%)	
rehabilitation facility	3 (3.8%)	2 (2.2%)	

Legend: Data are presented as median [IQR] or n (%)

*CPR* Cardiopulmonary resuscitation, *ICU* Intensive care unit, *ImCU* Intermediate care unit, *IQR* Inter quartile range, *LoS* Length of stay, *MR* Mitral regurgitation, *TR* Tricuspid regurgitation

### Cardiopulmonary resuscitation and unplanned transfer to ImCU/ICU

In the streamlined cohort, nine patients were admitted to either the ImCU or ICU following the intervention, while two patients were hypotonic after the procedure and required low doses of catecholamines. Two cases of cardiopulmonary resuscitation were observed: one patient suffered an iatrogenic mechanicly induced atrio-ventricular block III° due to transseptal puncture, while the other patient had a gastric puncture with major bleeding. Following a brief period of cardiopulmonary resuscitation and emergency surgical intervention (gastric resection), the patient was transferred to the ICU. During the operation, it was observed that venous bypass circuits, likely the result of a splenectomy performed 72 years ago, were bleeding heavily. The patient passed away the following day. Another patient exhibited signs of disorientation in the holding area and was transferred to the ImCU. On arrival, the patient was fully orientated and promptly admitted to the GW. Following the intervention, an additional patient was monitored in the ImCU after an incidental thoracic aortic aneurysm was identified during the procedure. The occurrence of a haematoma at the access site resulted in another patient being monitored on the ImCU. One patient exhibited symptoms indicative of hypertensive lung oedema and was administered non-invasive ventilation at the ImCU. The symptoms of dyspnoea and tachycardia were rapidly abated, and the patient was subsequently transferred to the GW. During the course of the procedure, one patient suffered a ventricular perforation, necessitating emergency cardiac surgery. Afterwards, the patient was monitored on the ICU.

### Clinical outcomes

At discharge, no differences were observed in the safety outcomes between the study groups (Table 4). Although more patients in the ICU/ImCU group developed acute kidney injury (ICU/ImCU: 4.4% vs. streamlined: 0%), the difference did not reach statistical significance (*p* = 0.064).

**Table 4:** Clinical outcomes according to MVARC criteria

	Discharge			30 days			6 months			12 months			
	ICU/ImCU **(*n*** **= 113)**	Streamlined **(*n*** **= 95)**	*p*-value	ICU/ImCU **(*n*** **= 108)**	Streamlined **(*n*** **= 92)**	*p*-value	ICU/ImCU **(*n*** **= 99)**	Streamlined **(*n*** **= 84)**	*p*-value	ICU/ImCU **(*n*** **= 97)**	Streamlined **(*n*** **= 79)**	*p*-value	
Loss to follow-up	**-**	**-**	**-**	4 / 113	z 3 / 95	> 0.9	14/113	11/95	0.9	16/113)	17/95	0.5	
All-cause mortality	0 (0)	1 (1.0)	0.5	1/108(0.9)	2/89 (2.2)	0.6	10/99(10.1)	4/84 (4.7)	0.2	16/97(16.0)	9/78 (12.0)	0.4	
Stroke	1 (0.9)	0 (0)	> 0.9	-	-	-	-	-	-	-	-	-	
TIA	0 (0)	0 (0)	> 0.9	-	-	-	-	-	-	-	-	-	
Delirium	1 (0.9)	1 (1.0)	> 0.9	-	-	-	-	-	-	-	-	-	
Major access complication	0 (0)	0 (0)	> 0.9	-	-	-	-	-	-	-	-	-	
Major vascular complication	0 (0)	1 (1.0)	0.5	-	-	-	-	-	-	-	-	-	
Major bleeding (Type 3 and 4)	0 (0)	2 (2.1)	0.2	-	-	-	-	-	-	-	-	-	
AKI	5 (4.4)	0 (0)	0.064	-	-	-	-	-	-	-	-	-	
Arrhythmia and conduction system disturbance (new AF)	0 (0)	0 (0)	-	-	-	-	-	-	-	-	-	-	
Device embolization	1 (0.9)	0 (0)	> 0.9	1/101 (1.0)	0/87(0)	> 0.9	-	-	-	-	-	-	
Thrombosis	1 (0.9)	0 (0)	> 0.9	1/101 (1.0)	0/87 (0)	> 0.9	-	-	-	-	-	-	
Single leaflet device attachment	2 (1.8)	3 (3.2)	0.7	2/101 (0)	3/87 (3.4)	0.7	-	-	-	-	-	-	
Heart surgery	0 (0)	1 (1.0)	0.5	0/104 (0)	1 / 88 (1.1)	0.5	1/80 (1.3)	1/71 (1.4)	> 0.9	-	-	-	
Reintervention	0 (0)	0 (0)	> 0.9	2/104(1.9)	0/89(0)	0.5	3/79 (3.8)	1/72 (1.4)	0.6	-	-	-	
Hospitalisation													
All-cause	-	-	-	-	-	-	35/79 (44)	24/73 (33)	0.15	-	-	-	
For heart failure	-	-	-	-	-	-	17/79 (22)	14/73 (19)	0.7	-	-	-	

Legend: Data are presented as n (%) or n/N (%)

*AF* Atrial fibrillation, *AKI* Acute kidney injury, *ICU* Intensive care unit, *ImCU* Intermediate care unit, *MVARC* Mitral Valve Academic Research Consortium, *TIA* Transient ischaemic attack

At 30 days, no major changes in complication rates were observed compared to the discharge timepoint. Notably, three more patients (one in the ICU/ImCU group and two in the streamlined group) died during this period. In the ICU/ImCU group, two patients (1.9%) underwent early reintervention with a new strategy, due to an unsuccessful first intervention.

**Fig. 3:**
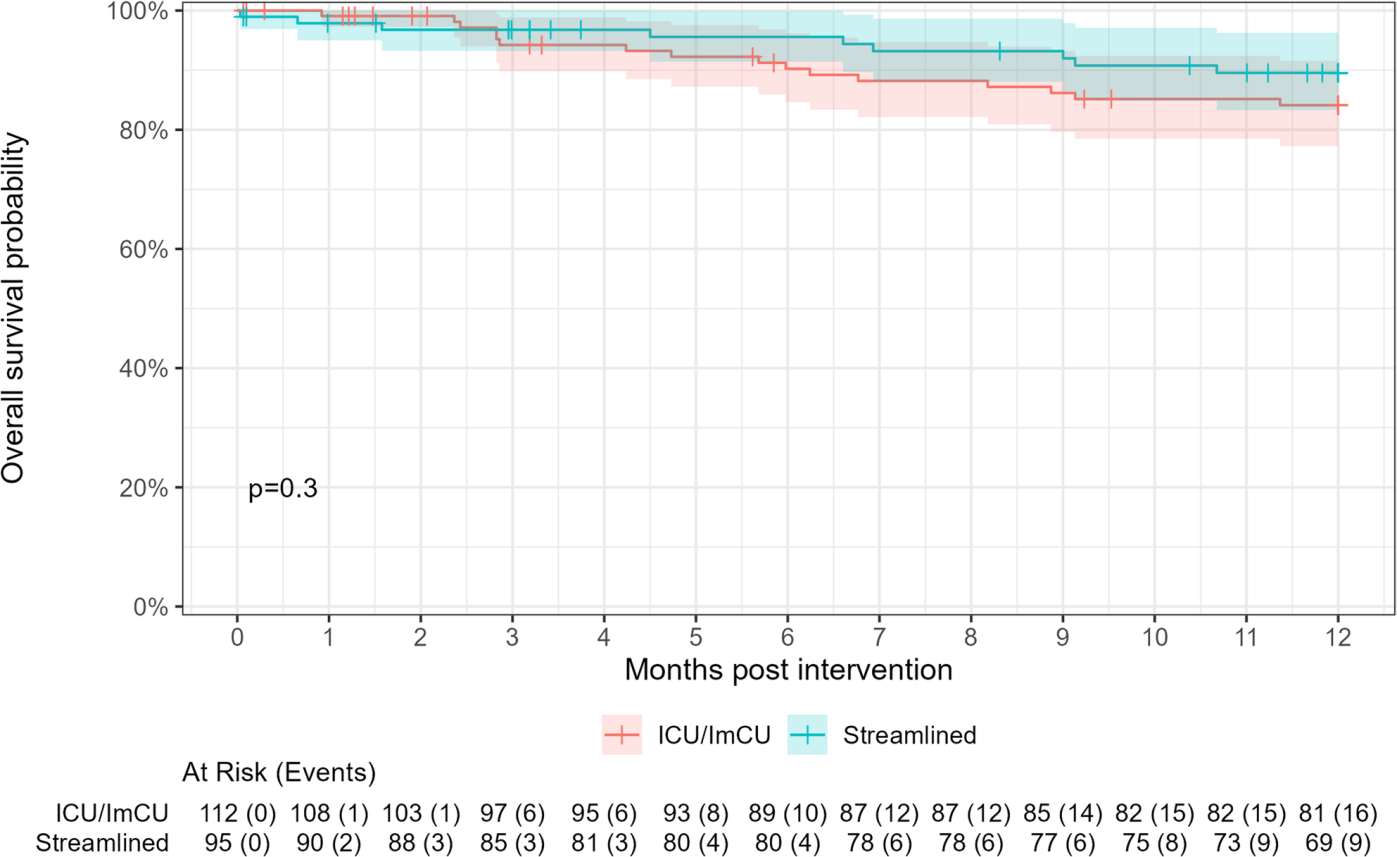
Overall survival at 12 months. Legend: ICU, intensive care unit; ImCU, intermediate care unit

At 6-month follow-up, reliable data on mortality, reintervention and hospitalization were available. In addition, there was no evidence for higher complication rates in the streamlined group: heart surgery (ICU/ImCU: 1.3% vs. streamlined: 1.4%, *p* > 0.9) and reintervention (ICU/ImCU: 3.8% vs. streamlined: 1.4%, *p* = 0.6), mortality was numerically higher in the ICU/ImCU group (ICU/ImCU: 10.1% vs. streamlined: 4.7%), but did not reach statistical significance (*p* = 0.2; Table 4; Fig. 3). There was a high rate of rehospitalisation (all-cause) in both groups, although the rate was numerically lower in the streamlined group (streamlined: 33% vs. ICU/ImCU: 44%) without being statistical significant (*p* = 0.15). Nonetheless, this difference is of clinical relevance. The rates of heart failure hospitalisation were comparable between the study groups (ICU/ImCU: 22% vs. streamlined: 19%, *p* = 0.7).

Similarly to discharge, 30-day, and six-month follow-up, at 12 monts both groups displayed comparable indications for all-cause mortality (ICU/ImCU: 16.0% vs. streamlined: 12.0%, *p* = 0.4).

## Discussion

In this retrospective, single centre analysis of a new streamlined postprocedural care algorithm after M-/T-TEER, the following key findings were identified: (1) The transition to a postprocedural care on a general cardiology ward was not observed to be associated with an elevated mortality and/or complication rate. (2) A statistically significant reduction in both the total and postprocedural length of stay was observed in the streamlined group in comparison with the ICU/ImCU group. (3) Patients in the streamlined group tended to have fewer all-cause hospitalisations at 6 months.

The hypothesis that an ICU/ImCU ward has negative effects on patients and should be avoided whenever possible can be derived from the various hospitalization-associated complications, in particular in the elderly [17, 18]. Delirium is a recognized complication in the ICU, especially in patients with multiple comorbidities and advanced age [19]. As the management of ICU delirium has been historically challenging, prompt mobilization is one of the few interventions demonstrated to reduce the risk of delirium [20]. Furthermore, the results of a large prospective study of over 44,000 patients in 27 countries demonstrated higher mortality rates among patients admitted directly to critical care services following elective surgery, even after risk adjustment, identifying no beneficial effect of critical care admission [21]. Maluangnon et al. also reported higher 28-day mortality rates among critically ill patients in ICU compared to those on a GW, concluding that mortality was strongly associated with disease severity without any additional mortality benefits of ICU admission [22]. Ziser et al. reported the outcomes of patient overflow admission to the post-anaesthesia care unit after surgery due to shortage of beds in the ICU facilities and showed that around 90% of patients were discharged within 24 h with a mean length of stay of 12.9 h, which indicates that ICU admission may not always be necessary [23]. Another benefit of providing immediate care to patients on GW rather than on ICU/ImCU is the reduction of costs by optimising nurse-to-patient ratios, since resources are frequently limited [24]. Thus, if GWs are filled with adequate staffing of qualified nurses, this would lead to a more cost-effective treatment in terms of post-surgical outcomes (reduced mortality rates), particularly in high-risk patients.

Cardiac surgical patients generally occupy a high proportion of beds in the critical care units due to the detrimental effects of cardiopulmonary bypass and prolonged anaesthesia. The fact that optimizing and streamlining the hospital stay associated with cardiac interventions frees up resources, increases patient safety and satisfaction without increasing complication rates is obvious, but has not often been proven in studies or retrospective investigations. So far, this has been demonstrated most impressively with transcatheter aortic valve implantation (TAVI): streamlining postprocedural care, including early mobilization and early discharge, was found to be safe and feasible [25–27]. For TEER procedures, the evidence is limited to retrospective studies in the field of M-TEER that compare the postprocedural care on ImCU to the care on specialised “valve units”. A decade ago, Di Prima et al. had already postulated, based on their observation on uneventful stays on ICU and fast admission of M-TEER patients to a GW, that a routine ICU admission might not be necessary for all patients [28]. Recently, Gröger et al. showed in a retrospective single centre analysis from Germany, that the admission to a dedicated “valve unit” was not inferior to a ICU admission with 84% of patients receiving adequate care in such units, although no beneficial effects could be demonstrated [7]. Similarly to our study, total hospital LoS was significantly shorter in the valve unit group as compared to those admitted to ICU/ImCU (median 6.0 vs. 7.0 days, *p* < 0.001) [7]. However, in addition to the shorter hospital LoS and non-inferiority of a streamlined approach compared to the ICU/ImCU admission in our study, a numerically lower number of patients who were admitted directly to the GW were hospitalised at 6 months. In contrast to the study by Gröger et al., the present study included all TEER patients (both M-TEER and T-TEER) in the study cohort and patients were followed for 12 months, whereas patients in the Gröger et al. cohort were not followed after discharge. Furthermore, patients in the streamlined group of study were admitted to a general cardiology ward as opposed to a heart valve unit.

Despite the fact that all patients in our cohort were treated with GA, the streamlining of the postprocedural process was successful. It has been previously reported that perioperative hypotension, which is typically observed in patients undergoing GA, and duration of anaesthesia are associated with complications and prolonged hospital stay [29]. In a recent meta-analysis comparing deep sedation and GA for M-TEER, the LoS in the ICU was also reported to be longer in the latter group [30]. However, the authors noted that this was driven primarily by the fact that most patients treated with DS were not admitted to the ICU at all (i.e., zero days on ICU) and not due to the associated complications. In fact, one may even speculate that the use of GA has had a certain role in the effective streamlining of the postprocedural care, due to lower risk of infection and the need for emergency intubation. Nonewithstanding, the findings of this study demonstrate that GA with extubation immediately in the operating room does not invariably necessitate ICU admission in a considerable proportion of patients, thereby corroborating the observations reported by Gröger et al. [7].

Overall, the advantages of omitting ICU/ImCU care in the present and previous studies along with prospective reduction in capital expenditure and manpower requirements should be considered in order to formulate comprehensive structural heart disease programs and hospital models that can deliver the highest level of care to patients undergoing TEER.

## Limitations

This is a retrospective single centre study with its known limitations and susceptibility to bias. The pseudo-randomisation through group formation at a date cut-off, which was due to administrative reasons, attempts to counteract this. However, there remained a difference in the number of patients with HFrEF, favoring the streamlined group, which could have influenced the results. Further limitations arise by the significant loss to follow-up, in particular at the 6-month and 12-month time points. The relatively small number of patients included in the study does not permit for meaningful subgroup analysis or the identification of patients who would benefit more from ICU/ImCU care based on a preoperative risk profile. Nevertheless, the findings in our study further encourage the conduct of a multicentre randomised trial.

## Conclusion

This study indicates that there are no significant patient safety concers associated with a change in postprocedural care, as no complications were identified that would have been preventable through admission to the ICU/ImCU. Moreover, the data suggest that a GW admission could positively influence patient outcomes by mitigating factors associated with delirium and promoting patient autonomy. In conclusion, the aforementioned modification has the potential to reduce the demand for nursing and ICU beds, thereby decreasing the financial burden on the health care system. This, in turn, could facilitate a more efficient utilization of resources.

## Data Availability

The datasets used and/or analysed during the current study are available from the corresponding author on reasonable request.

## Abbreviations

GA: General anaesthesia
GW: General ward
ICU: Intensive care unit
ImCU: Intermediate care unit
IQR: Interquartile range
LoS: Length of stay
M-/T-TEER: Mitral/tricuspid transcatheter edge-to-edge repair
MR: Mitral regurgitation
M-TEER: Mitral transcatheter edge-to-edge repair
NYHA: New York Heart Association
SD: Standard deviation
TEER: Transcatheter edge-to-edge repair
TR: Tricuspid regurgitation
T-TEER: Tricuspid transcatheter edge-to-edge repair
